# A Cross‐Sectional Survey of the Use of Clear Aligners by General Dentists in Australia

**DOI:** 10.1002/cre2.919

**Published:** 2024-07-07

**Authors:** Maurice J. Meade, Tony Weir

**Affiliations:** ^1^ Orthodontic Unit, Adelaide Dental School The University of Adelaide Adelaide South Australia Australia

**Keywords:** clear aligner therapy, general dentist, Invisalign, questionnaire, survey

## Abstract

**Objectives:**

The primary aim of the investigation was to survey clear aligner therapy (CAT) use among general dentists in Australia. A secondary aim was to evaluate the factors that influenced general dentists in Australia not to provide CAT.

**Material and Methods:**

General dentists registered with the Australian Health Practitioner Regulation Agency were invited to participate in a structured cross‐sectional electronic survey. The survey covered demographics, preferred CAT systems and practices, relevant treatment planning and retention protocols, patient‐reported CAT issues, pertinent respondent opinions, and reasons for not providing CAT. Descriptive statistics were computed via GraphPad Prism v10 (GraphPad Software Inc., La Jolla, CA, USA).

**Results:**

Most of the 264 (*n* = 172; 65.2%) respondents indicated that they provided CAT. The majority (*n* = 82; 58.6%) reported that they treated between 1 and 20 patients with CAT annually. Invisalign was the most used system (*n* = 83; 61.2%), with 55 (41.7%), indicating that they used more than one system. Most (*n* = 124; 98.4%) were comfortable using CAT for mild crowding, whereas 73.4% (*n* = 94) were not comfortable in treating severe crowding with CAT. The median (IQR) number of patients per respondent treated with extraction of a permanent incisor or premolar was 0 (0). Issues regarding tooth positions were reportedly always or mostly in need of change in the initial treatment plan by 68.7%. Problems regarding patient compliance with CAT wear protocols (*n* = 67; 45.6%) and the predictability of treatment outcomes (*n* = 31; 21.1%) were the most identified themes of the free‐text comments. Over 80% of those who did not provide CAT indicated that they preferred to refer to an orthodontist for management.

**Conclusion:**

Almost two‐thirds of the respondents provided CAT. Invisalign was the most used system. The majority use CAT combined with nonextraction treatment. Most of those who did not provide CAT preferred to refer to an orthodontist for patient management.

## Summary

- The use of CAT is increasing globally.
- The present survey is among the first to explore CAT practices of general dentists. As such, it provides novel baseline information related to a wide range of factors related to CAT practices and protocols.
- In the absence of robust evidence regarding CAT, it informs clinicians about colleagues' use, experiences, and problems with, and opinions of, CAT.
- The study will enable clinicians to provide insight regarding their own utility of CAT by comparing their own practices to the study's findings.

## 1 Introduction

The provision of clear aligner therapy (CAT) has been increasing globally over the past two decades (Vlaskalic and Boyd 2002). The proposed advantages of CAT such as its aesthetic appearance, easy removal for oral hygiene purposes, and the ability to manage treatment progress remotely have made it appealing to patients and clinicians alike (Lam Freer and Miles 2023).

CAT generally entails the formulation of a digital treatment plan (DTP) by the treating clinician via a digital interface (Meade, Ng, and Weir 2023). The DTP may incorporate the planned use of interproximal reduction (IPR) of enamel and/or the bonding of composite resin (CR) attachments of varying geometric shapes onto the labial/buccal surfaces of one or more teeth to aid the achievement of treatment objectives (Meade and Weir 2023a). Once the clinician is satisfied with the DTP, a series of plastic aligners is fabricated by the company supplying the CAT service. Each aligner is “programmed” to gradually move the teeth into the positions planned via the interface, with the usual objective that the achieved treatment outcome matches the planned treatment outcome at the last stage of wear of the final aligner in the series.

However, the treatment outcome planned in the initial DTP is rarely achieved (Bowman et al. 2023; Lim, Weir, and Meade 2023; Stephens et al. 2022). Consequently, one or more additional series of aligners and/or combined CAT treatment with fixed appliances are commonly required (Kravitz et al. 2023; Meade and Weir 2022). Suggested reasons for the planned and achieved outcomes not matching include suboptimal treatment planning, aligner company software deficiencies, aligner material shortcomings, and patient noncompliance with clinician‐prescribed wear protocols (Meade, Blundell, and Weir 2024; Meade, Ng, and Weir 2023; Timm et al. 2021, 2022).

Recent studies have investigated the CAT practices and protocols among specialist orthodontists in Australia and internationally (Abu‐Arqub et al. 2023; Meade and Weir 2022; Meade et al. 2023). Trends, such as the worldwide ubiquity of the Invisalign appliance and the acceptability among orthodontists of treating mild crowding and spacing cases, have been identified (Meade and Weir 2022; Meade and Weir 2023b; Meade et al. 2023).

Comparable information among general dentists in Australia and elsewhere, however, is limited. Although some studies have investigated aspects of CAT by general dentists in comparison with orthodontists, investigations focussing solely on general dentist CAT practices are few (Aacaashnathan et al. 2023; d'Apuzzo et al. 2019; Gao et al. 2024). Recent evaluation of the provision of orthodontic therapy by general dentists in Australia is limited to one study evaluating interceptive orthodontic practices in general practice (Currell Vaughan, and Dreyer 2019).

Although some information about general dentist CAT provision can be elicited from a study investigating the content of general dental practice websites, a detailed evaluation of CAT practices by general dentists is lacking (Meade and Dreyer 2022). The aim of the current cross‐sectional survey was to determine CAT practices among general dentists in Australia. A secondary aim was to evaluate the factors that influenced general dentists in Australia not to provide CAT.

## 2 Materials and Methods

The present investigation used the STROBE Statement checklist for cross‐sectional studies (Von Elm et al. 2014).

Ethical approval for this cross‐sectional investigation was granted by University of Adelaide Human Research and Ethics Committee (HREC‐2023‐031)

Participation in the investigation was voluntary and anonymous. Potential respondents were provided with written information before participation and were advised that submission of the questionnaire on completion indicated their consent to participate.

The questionnaire was adapted and modified from questionnaires used to determine CAT protocols among specialist orthodontists in Australia and elsewhere (Meade and Weir 2022; Meade et al. 2023). Four general dentists, practicing in Australia, assisted in validating the survey for the target audience and piloting indicated that completion of the questionnaire took less than 15 min.

The first of the questionnaire's nine parts concerned the respondents' demographic data. The second determined whether the respondents used CAT in their practice. Respondents who stated that they did not provide CAT to patients were requested in Part 9 to indicate to what level a predetermined list of factors influenced why they did not. Part 3 included questions on respondents' favored CAT systems. The following four parts comprised questions about DTPs, case selection, and CAT practices and protocols. Questions concerning post‐CAT retention appliances and respondent opinions concerning CAT were contained in Part 8.

The Australian Dental Association (ADA) is the largest representative body for dentists in Australia. Criteria for the inclusion of respondents were individuals who were registered as general dentists in Australian Health Practitioner Regulation Agency (AHPRA), and whose principal workplace, was in Australia. Exclusion criteria included dentists who were also registered as specialist dentists. In addition, non‐ADA members may have responded due to the nature of the dissemination of the survey. If these respondents otherwise satisfied the inclusion criteria, their responses were included for evaluation.

The questionnaire was developed on the SurveyMonkey (San Mateo, CA, USA) website. Information regarding the study and a link to the questionnaire was disseminated through federal and state ADA publications and social media platforms in addition to the evident Foundation Dental Practice‐Based Research Network. The survey was open from March 17 to September 30, 2023, and the utilization of cookies on web browsers by the website aimed to reduce the risk of repeat questionnaire submissions by the same individuals (Meade et al. 2023).

The questionnaire responses were captured on a Microsoft (version 16.0; Microsoft, Redmond, WA, USA) Excel spreadsheet where data cleaning was conducted before statistical analysis. Demographic statistics were computed via the GraphPad Prism (GraphPad Software Inc., La Jolla, CA, USA) statistics software facility. The results were expressed in frequencies and percentages and presented in text, tabular, and graphical form. Respondent comments were coded according to content into themes and subthemes, with comments frequently coded into more than one theme.

## 3 Results

After excluding the responses of three respondents who did not satisfy inclusion and exclusion criteria as they were specialist orthodontists, the responses from 264 general dentists were evaluated. The response rate was approximately 2%. Not all responded to every question and the percentage values provided are in relation to the number of respondents who answered the pertinent questions. Almost half of the respondents were male (*n* = 130; 49.3%) and half (*n* = 50.7%) were female.

The median (IQR) number of years in practice was 17 (8.25, 28.75; minimum 1, maximum 49). Most dentists obtained their dental degrees from universities in Australia (*n* = 210; 79.6%), the United Kingdom (*n* = 12; 4.6%), and Ireland (*n* = 6; 2.3%). The majority reported that they worked in private practice only (*n* = 230; 87.1%), followed by 16 (6.1%) respondents who worked in a community/hospital/university setting, and 14 (5.3%) community/hospital/university and private setting. Almost 66% (*n* = 174; 65.9%) of respondents reported that they carried out orthodontic treatment in their practice.

**Figure 1:**
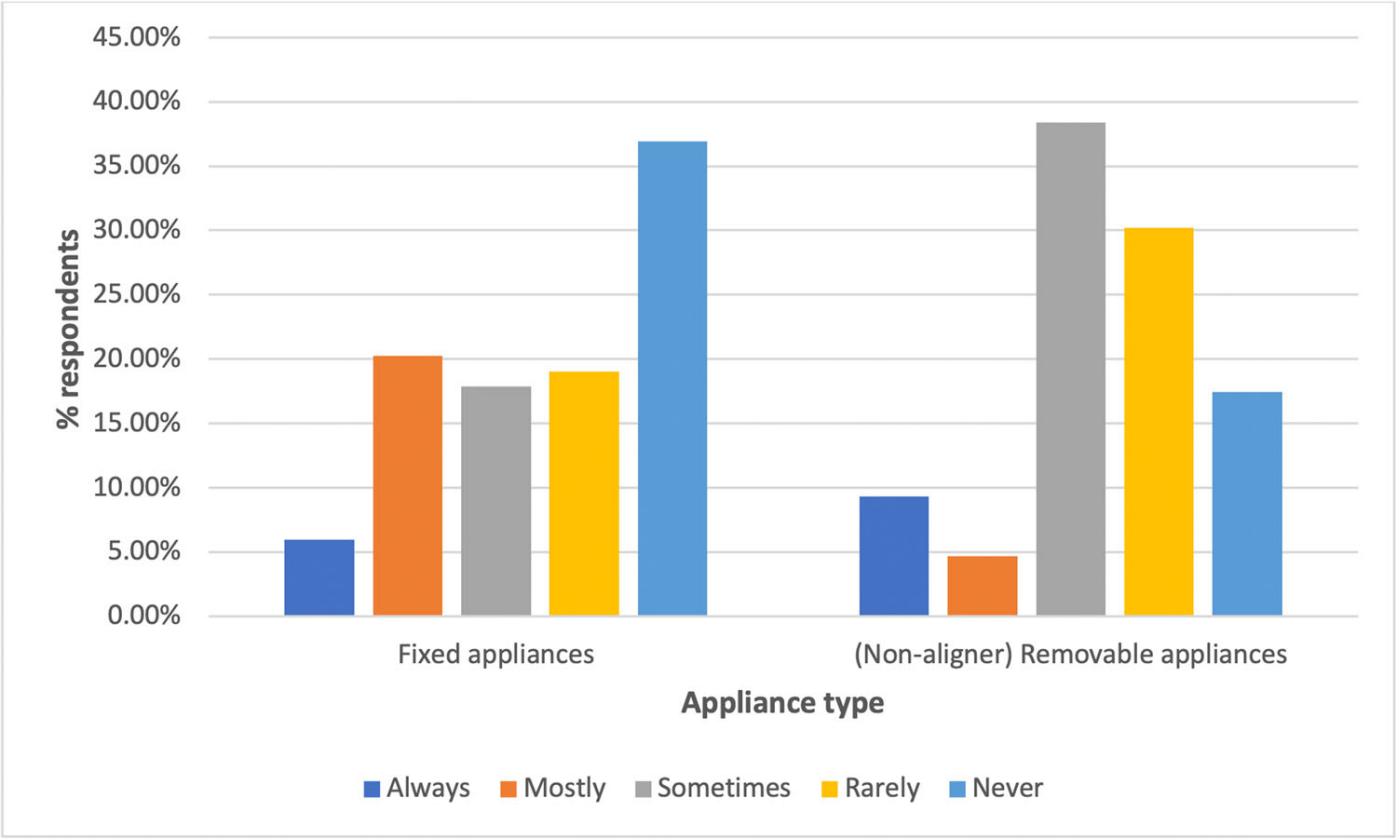
Frequency of use of fixed appliances and (nonaligner) removable appliances among respondents who reported the use of orthodontic appliances in practice (*n* = 174).

Figure 1 shows that 36.9% of respondents who carry out orthodontic treatment in their practice reported that they do not use fixed appliances.

Of the 174 who reported that they carried out orthodontic treatment in their practice, 172 (98.9%) indicated that they used CAT. The respondents reported that the median (IQR) approximate percentage of their annual orthodontic case load that was treated with CAT was 90 (30, 100). The respondents reported that treated a median (IQR) of 0 (0, 0) patients in the mixed dentition, a median of 0 (0, 2) adolescent/teenage patients, and a median of 6 (2, 25.8) in the adult dentition.

Table 1 shows that most respondents considered that they treated between 1 and 20 patients with aligners each year. Table 2 outlines the aligner systems used by the respondents. Fifty‐five (41.7%) indicated that they used more than one system, with the ClearCorrect (Straumann Holding AG, Basel, Switzerland) system being used by almost 20% of respondents. However, the Invisalign (Align Technology, San Jose, CA, USA) appliance was the system most commonly used by the respondents.

**Table 1:** Approximate number of patients treated with aligners by respondents per year (*N* = 141).

	Respondent	
Patients treated with aligners per year (*N*)	*N*	%
1–10	51	36.2
11–20	32	22.7
21–50	28	19.9
51–100	18	12.9
101–200	8	5.7
> 200	4	2.9

Abbreviations: N, number; >, greater than; %, percentage.

**Table 2:** Aligner systems used by respondents (*N* = 132).

	A. Which of the following aligner systems do you use in your practice?		B. Which of the following aligner systems do you use in your practice the most?	
System	*N*	%	*N*	%
ClearCorrect	26	19.1	20	14.9
In‐house	10	7.4	0	0.0
Invisalign	100	73.5	82	61.2
SmileStyler	6	4.4	6	4.5
SmileTRU	0	0.0	0	0.0
Spark	24	17.7	12	9.0
Truline	0	0.0	0	0.0
AngelAlign	28	21.2	8	6.1
Other	10	35.3	0	0.0

*Note:* Please note that respondents can give more than one option in question A. AngelAlign: Wuxi Angel Align Biotechnology Co., Wuxi, China; ClearCorrect: Straumann Holding AG, Peter Merian‐Weg 12, 4002 Basel Switzerland; Invisalign: Align Technology, San Jose, CA, USA; SmileStyler: Unit 1, 40‐44 Cook Street, Port Melbourne, VIC 3207 Australia; SmileTRU: PO Box 1419 Gainesville Texas 76240, USA; Spark: Spark Ormco Corporation: Orange, CA, USA; Truline: 900 Botany Road, Mascot, New South Wales 2020, Australia.

Table 3 indicates that patient satisfaction is a major or moderate influence for 82.6% of respondents in the decision to use a particular aligner company. Almost all (*n* = 130; 97%) respondents reported that they made changes to the DTP proposed by the aligner system before accepting it. Most (*n* = 96; 71.7%) reported making an average of one to three changes before acceptance, with 28 (20.9%) respondents indicating that they made four to six changes and 6 (4.5%) considering that they made more than six changes before acceptance.

**Table 3:** Factors that influence the decision to use a particular company/provider.

			Influence								
			No		Minor		Moderate		Major		
			*N*	%	*N*	%	*N*	%	*N*	%	Total, *N*
Cost			24	17.4	42	30.4	46	33.3	26	18.9	138
Ease of digital treatment planning			12	8.7	10	7.3	54	39.1	62	44.9	138
Aesthetics of appliances			18	13.0	22	16	46	33.3	52	37.7	138
Patient satisfaction			10	7.3	14	10.1	44	31.9	70	50.7	138
Brand identity			40	29	40	29.0	26	18.9	32	23.2	138
Ongoing education by provider			42	30.4	42	30.4	34	24.6	20	14.5	138
Assistance in troubleshooting			22	16.0	46	33.3	46	33.3	24	17.4	138
Corporate support in advertising			60	43.5	36	26.1	30	21.7	12	8.7	138

Abbreviations: N, number; %, percentage.

**Figure 2:**
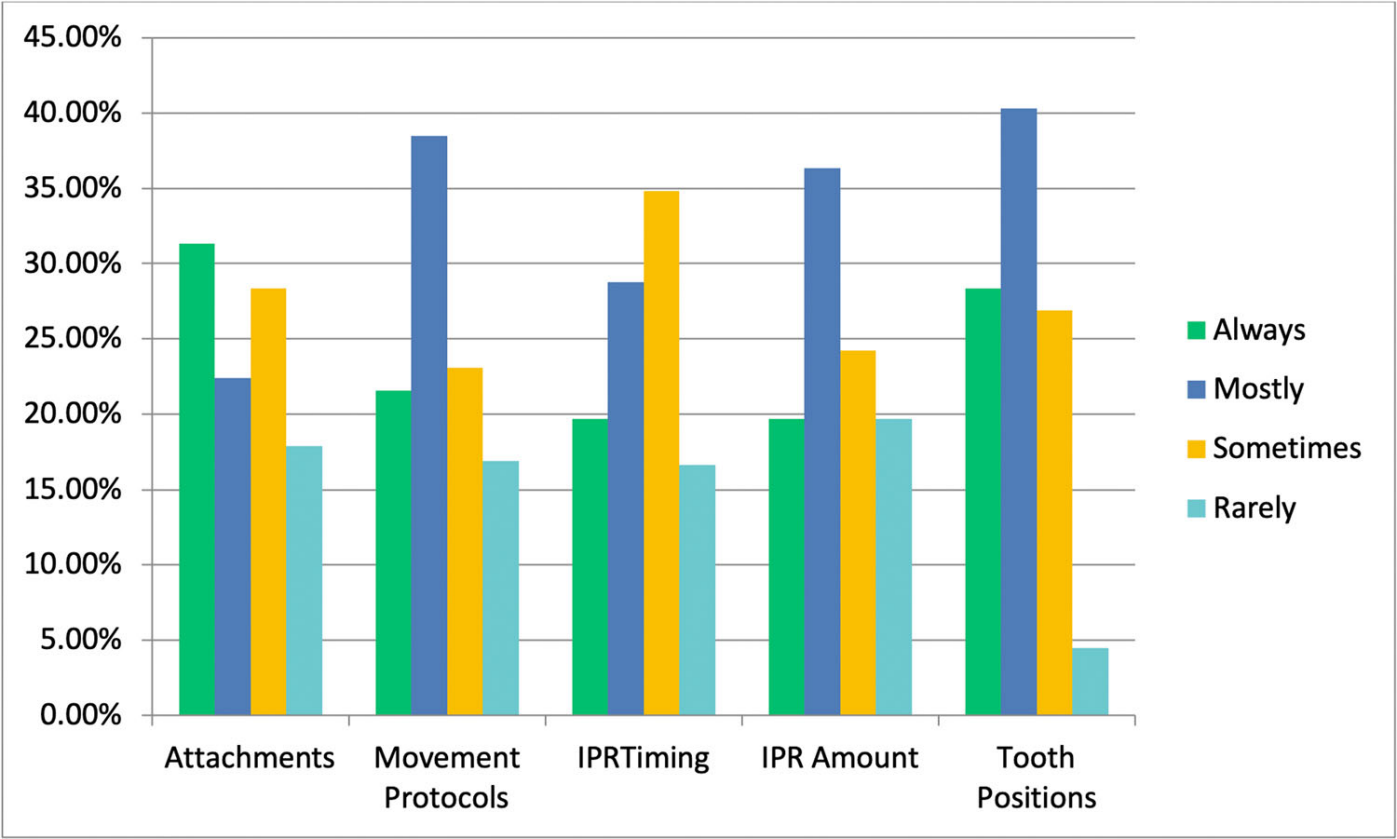
Areas reported by respondents most in need of changes from the initial DTP received from the aligner provider (*n* = 134). IPR, interproximal reduction.

Figure 2 shows that almost 70% of respondents considered “tooth positions” to be an area that was always or mostly in need of change from the initial DTP received from the aligner provider. Ten (7.5%) respondents indicated that they always used the services of a third party to assist with DTP, whereas 36 (26.9%) reported that they mostly or sometimes used a third party. Eighty‐eight (65.5%) responded that they rarely or never used the services of a third party.

Table 4 shows that most respondents are comfortable in treating mild crowding cases and the majority are uncomfortable in treating cases with severe crowding with CAT, either with or without extractions. Eight (6.3%) respondents reported that they carried out premolar extractions in combination with CAT, whereas 26 (21.3%) reported that they had carried out lower incisor extraction cases combined with CAT. Twenty‐eight (22.6%) respondents indicated that they used bite ramps in their deep bite cases and 13 (10.5%) reported that they used CAT combined with temporary anchorage devices. The median (IQR) number of patients per respondent treated with each of the above modalities was 0 (0).

**Table 4:** Comfort in treating different treatment situations (with or without extractions).

	Comfortable		Uncomfortable	
Situation	*N* = 126		*N* = 128	
	*N*	%	*N*	%
↑ overjet	70	55.6	22	17.2
↓ overjet	48	38.1	28	21.9
↑ overbite	44	34.9	72	56.3
↓ overbite	70	55.6	22	17.2
Posterior x‐bite (unilateral)	44	34.9	56	43.8
Posterior x‐bite (bilateral)	20	15.9	78	60.9
Spaced dentition	98	77.8	8	6.3
Mild crowding (0–4 mm)	124	98.4	2	1.6
Moderate crowding (4.1–8 mm)	76	60.3	26	20.3
Severe crowding (> 8 mm)	20	15.9	94	73.4
Other	8	6.4	20	15.6

Abbreviations: N, number; x‐bite, crossbite; ↑, increased; ↓, decreased.

Dental monitoring or another remote monitoring system was reportedly always (*n* = 20; 15.6%) or mostly (*n* = 16; 12.5%) used by over a quarter of respondents, whereas over half never (*n* = 74; 57.8%) or rarely (*n* = 6; 4.7%) used any remote monitoring system.

The respondents recommended changing aligners fortnightly in 50.9% (*n* = 58) of adult cases and 33.9% (*n* = 42) of adolescent/teenage cases, whereas a weekly change was recommended in 45.6% (*n* = 52) of adult cases and 40.3% (*n* = 50) of adolescent/teenage cases. Eighteen respondents (14.3%) indicated that they recommended a change of aligners every 10 days.

Table 5 shows that respondents required patients to attend progress checks every 6 weeks most commonly.

**Table 5:** Frequency of progress checks per respondent (*N* = 128).

Frequency	*N*	%
Every 4 weeks	20	15.6
Every 6 weeks	42	32.8
Every 8 weeks	30	23.4
Every 12 weeks	14	10.9
Only if there is a problem, otherwise I provide all the aligners and review at the end of treatment or when remote monitoring advises	8	6.3
Other	14	10.9

Abbreviations: N, number; %, percentage.

A median (IQR) of 75% (50, 90) of cases was reported by the respondents to have had IPR prescribed in the initially accepted plan, whereas a median (IQR) of 20% (10, 50) of cases was reported by the respondents to have had IPR prescribed in the additional/refinement plan.

Over half (*n* = 62; 50.8%) reported that the prescribed IPR was routinely less in the additional plans or both more and less (*n* = 30; 24.6%) in the additional aligner/refinement plans compared with the initial plans.

**Figure 3:**
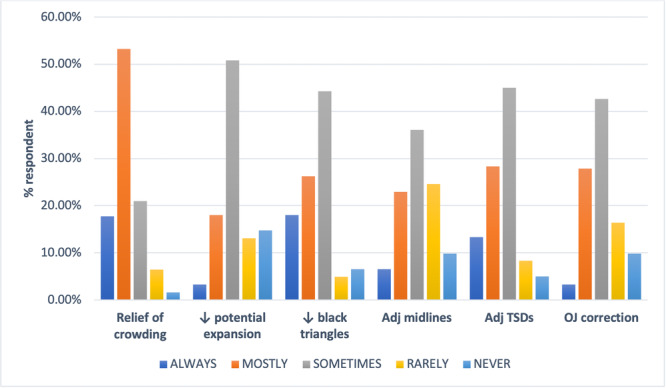
Frequency of prescription of IPR to address treatment objectives (*n* = 124). Adj, adjustment; OJ, overjet; TSD, tooth size discrepancy; ↓, reduction.

Figure 3 indicates that IPR was always or mostly prescribed (*n* = 88; 71%) in respondents' initial DTPs to relieve crowding.

Table 6 shows that solid and perforated strips were most commonly used to carry out IPR by the respondents.

**Table 6:** Tools used to perform IPR in CAT.

	Always		Mostly		Sometimes		Rarely		Never		
	*N*	%	*N*	%	*N*	%	*N*	%	*N*	%	Total, *N*
Perforated strip	24	20.3	32	27.1	22	18.6	18	15.3	22	18.6	108
Solid strip	26	22.4	46	39.7	26	22.4	10	8.6	8	6.9	106
Perforated disc	2	1.9	4	3.7	4	3.7	22	20.4	76	70.4	108
Solid disc	2	1.9	8	7.6	4	3.8	22	20.8	70	66.0	106
High‐speed bur	2	1.8	16	14.3	30	26.8	18	16.0	46	41.1	102
Oscillating disc	0	0.0	2	1.8	4	3.6	8	7.3	96	87.3	110
Reciprocating strip	4	3.6	6	5.5	14	12.7	18	16.4	68	61.8	110

Abbreviations: CAT, clear aligner therapy; IPR, interproximal reduction; N, number; %, percentage.

The median (IQR) % number of their annual caseload estimated by respondents to have one or more additional series was 87.5 (60, 99) and the reported median (IQR) number of additional series of aligners was 1.5 (1, 2). The median (IQR) average treatment time per dual arch nonextraction CAT case reported by the respondents was 14 (10, 18) months.

Table 7 indicates that a thermoplastic retainer only in the maxilla and a combined BR and TR in the mandible were most commonly prescribed after CAT by respondents. Table 8 outlines the frequency of patient‐reported aligner‐related issues as reported by the respondents.

**Table 7:** Most common retainer prescribed following CAT (*N* = 118).

	Max		Man	
Retainer	*N*	%	*N*	%
BR only	18	15.3	26	22.0
TR only	70	59.3	30	25.4
BR and TR	24	20.3	59	50.0
BR and Hawley	1	0.9	2	1.7
None	0	0.0	0	0.0
Other	5	4.2	1	0.9

Abbreviations: BR, bonded retainer; CAT, clear aligner therapy; Man, mandible; Max, maxilla; N, number; TR, thermoplastic retainer; %, percentage.

**Table 8:** Frequency of patient‐reported aligner‐related issues by respondent (*N* = 118).

	Frequency										
	Always		Mostly		Sometimes		Rarely		Never		
Issue	*N*	%	*N*	%	*N*	%	*N*	%	*N*	%	Total, *N*
Appearance of aligner	0	0.0	2	1.7	8	6.9	56	48.3	52	43.1	116
Appearance/discomfort of attachments	0	0.0	2	1.7	30	25.9	66	56.9	18	15.5	116
Difficulty in compliance with wear protocol	4	3.4	4	3.4	58	49.2	46	39.0	6	5.1	118
Discomfort	2	1.7	10	8.6	44	37.9	54	46.6	6	5.2	116
Dissatisfaction with treatment length	0	0.0	2	1.7	48	41.4	46	39.7	20	17.2	116
Dissatisfaction with treatment outcome	0	0.0	2	1.7	18	15.5	74	63.8	22	19.0	116
Dissatisfaction with a need for refinement	0	0.0	4	3.4	32	27.1	60	50.9	22	18.6	118
Loss of aligner	0	0.0	2	1.7	24	20.7	84	72.4	6	5.2	116
Poor fit of aligner(s)	0	0.0	6	5.2	32	27.6	66	56.9	12	10.3	116
Speech/lisping	0	0.0	8	6.9	44	37.9	52	44.8	12	10.3	116
Staining of teeth around attachments	0	0.0	12	10.3	36	31.0	52	44.8	16	13.8	116
Wear/breakage of aligner	0	0.0	2	1.7	30	25.9	72	62.1	12	10.3	116

Abbreviations: N, number; %, percentage.

Forty‐two (36.2%) respondents agreed (either strongly or somewhat) that matched cases achieving identical outcomes treated with aligners take longer compared with fixed appliances, whereas 26 (22.4%) disagreed (either strongly or somewhat). The remainder (48; 41.4%) neither agreed nor disagreed. They reported that both take the same time.

Twelve (10.3%) respondents agreed (either strongly or somewhat) that matched cases treated with aligners produce superior outcomes compared with fixed appliances, whereas 54 (46.5%) disagreed (either strongly or somewhat). The remainder (*n* = 50; 43.1%) neither agreed nor disagreed. They reported that both have similar outcomes.

Table 9 indicates that over 80% of respondents considered that a preference for the orthodontist to manage the patient was a moderate or major influence on the decision not to use CAT. Table 10 outlines the themes and subthemes into which the respondent comments were coded. Issues regarding patient compliance with CAT wear protocols (*n* = 67; 45.6%) and the predictability of treatment outcomes (*n* = 31; 21.1%) were the most identified themes.

**Table 9:** Factors that determine the decision not to use CAT.

	Influence								
	No		Minor		Moderate		Major		
Factor	*N*	%	*N*	%	*N*	%	*N*	%	Total, *N*
My practice environment (e.g., university) does not provide it	41	50.0	11	13.4	10	12.2	22	26.8	82
Other clinicians in my practice provide aligner treatment	47	58.8	17	21.3	6	7.5	10	12.5	80
Initial costs to the practice	55	68.8	17	21.3	2	2.5	6	7.5	80
Ongoing costs to the practice	56	70.0	18	22.5	2	2.5	4	5.0	80
Costs to the patient	51	62.3	16	19.8	7	8.6	7	8.6	81
Dependency on a third party for tx provision	37	46.3	19	23.7	12	15	12	15.0	80
“Fixed appliances provide better tx outcomes”	34	42.5	19	23.8	19	23.8	8	10.0	80
Patient expectations	33	40.8	18	22.2	14	17.3	16	19.8	81
Concerns over patient compliance with tx protocols	31	38.8	25	30.9	13	16	12	14.8	81
Insufficient postgraduate education	13	16.25	20	25.0	10	12.5	37	46.3	80
Concerns over ongoing aligner education	24	30.0	23	28.8	21	26.3	12	15.0	80
Prefer to refer to orthodontist for patient management	9	11.1	5	6.2	18	22.2	49	60.5	81

Abbreviations: CAT, clear aligner therapy; tx, treatment.

**Table 10:** Frequency of respondent comments in each theme and subtheme (*N* = 147).

Theme	Subthemes	*N*	%
Aesthetics		8	5.4
Extraction treatment		11	7.5
Compliance		67	45.6
Cost		10	6.8
Education		14	9.5
Treatment issues	Deep bite	12	8.2
	Rotations	8	5.4
	Torque/bodily movement	9	6.1
	Open bite	7	4.8
	Extrusion of teeth	8	5.4
	Intrusion	7	4.8
	Extraction/root tipping	5	3.4
	Finishing/results	9	6.1
Material		22	15
Predictability/unpredictability		31	21.1
Adjuncts	IPR	5	3.4
	Attachments	8	5.4
Treatment planning	Initial	12	8.2
	Refinement	12	8.2
	Tracking	11	7.5
Company support		7	4.8

Abbreviations: IPR, interproximal reduction; N, number; %, percentage.

## 4 Discussion

The current cross‐sectional study is the first to investigate CAT practices among general dentists. The findings indicated that almost two‐thirds of the surveyed dentists provided CAT to their patients, and those who did provide CAT provided predominantly nonextraction to adult patients. It found that Invisalign was the most commonly used appliance and a preference to refer to an orthodontist for orthodontic management was among the factors that influenced the nonuse of CAT by respondents.

The number of respondents in the present survey compared with 149 to 382 general dentists who responded to recent surveys conducted in Australia (Brennan et al. 2020; Currell, Vaughan, and Dreyer 2019; George et al. 2017; Teoh et al. 2019). It also compared with 43 to 87 general dentist respondents in studies conducted outside Australia and involving comparison with orthodontists (Aacaashnathan et al. 2023; d'Apuzzo et al. 2019; Gao et al. 2024). The dissemination of the questionnaire through ADA publications and social media outlets made the determination of the response rate challenging. It is not unreasonable, however, to conclude that the response rate was low (in the order of 2%) particularly as the number of general dentists registered with the Australian Health Practitioner Regulatory Agency, although likely to be greater than those who are members of the ADA, was 17,303 in September 2023 (https://www.dentalboard.gov.au/News/2023-11-09-quarterly-registration-data.aspx). The current survey aimed to capture as many respondents as possible so calculation of the sample size was not carried out. However, future surveys should consider random sampling, with appropriate precision, and utilizing strategies to encourage participation to minimize nonresponse error (Shelley and Horner 2021).

Males comprised 50.7% of the respondents which compared with the response rates from males of 48.9%–54%% recorded in recent investigations (George et al. 2017; Teoh et al. 2019). The median number of years in dental practice was 17.0 years, which was almost identical to the mean number of 17.2 years in specialist practice reported by specialist orthodontists in a recent survey in Australia (Meade and Weir 2022).

The Invisalign appliance was the most commonly used CAT appliance by the respondents. At 61.2%, it was very close to the 60.6% reported by respondents in the survey reporting CAT practices among orthodontists in Australia (Meade and Weir 2022). Interestingly, the findings of a study of 231 Australian general dental practice websites indicated that 69.3% of the websites contained information and/or links to the Invisalign appliance (Meade and Dreyer 2022). Brand identity was reported to be a major or moderate influence for 42% of the respondents in their decision to use a particular aligner in the present survey. Further research is required to determine the role of the internet and public awareness of orthodontic products and clinician decisions to use specific orthodontic products.

Most respondents indicated that they made changes to the initial DTP before they accepted the plan. This was consistent with the findings from surveys and retrospective investigations of DTP practices of specialist orthodontists in Australia and elsewhere (Meade and Weir 2023a; Meade, Ng, and Weir 2023; Meade et al. 2023). Furthermore, the respondents considered that a median of 87.5% of their caseload required one or more than one additional series of aligners and this aligned with the findings of 80%–90% among orthodontists in Australia and internationally (Meade and Weir 2022; Meade et al., 2023). The clinician's need to routinely change the DTP before acceptance emphasizes the crucial part the clinician plays in CAT treatment planning.

The majority of respondents who provide CAT reported that they were comfortable providing CAT for the management of mild crowding. This is understandable as the modern iteration of CAT was initially focused on addressing mild malalignment (Vlaskalic and Boyd 2002). In addition, the respondents reported that the prescribed extraction of permanent teeth in combination with CAT was uncommon. The median (IQR) number of patients per respondent treated with extraction of a permanent incisor tooth or premolar was zero. This contrasted with 58.8% of orthodontists in the United States and Canada who reported that they had “commonly” prescribed extraction of a lower incisor as part of CAT (Abu‐Arqub et al. 2023). This suggested that general dentists were more at ease managing milder malocclusions with CAT.

Just over half of the respondents recommended changing aligners in adult patients every fortnight. This differed from findings among orthodontists in Australia, the United States, and Canada where a weekly change protocol was most common (Abu‐Arqub et al. 2023; Meade and Weir 2022). However, the wide range of protocols reported in the present survey suggested that respondents may be advising an aligner change protocol tailored for the individual patient. Progress checks every 6 weeks were most frequently recommended by respondents. This differed from orthodontists in Australia, the United Kingdom, and the Republic of Ireland where the recommended interval was 8 weeks (Meade and Weir 2022; Meade et al. 2023). However, over a quarter always or mostly used a remote monitoring system that may influence how frequently patients will be required to attend the clinic for progress checks in the future (Lam, Freer and Miles 2023).

The evidence indicates that IPR performed as part of CAT is commonly less than prescribed (De Felice et al. 2020; Weir et al. 2021; Weir, Shailendran, and Kerr 2022). The respondents in the present survey reported that they prescribed IPR in a median of 75% of their patients in the initial DTP. This was greater than the 55% reported by respondents in the survey of orthodontists in Australia (Meade and Weir 2022). It was also greater than the median of 20% of patients reportedly prescribed IPR by the respondents in the additional aligner/refinement plan in the present survey. Respondents indicated that the procedure was always or mostly prescribed for the relief of crowding in over 70% of the initial DTPs. This contrasted with 51.2% in the corresponding survey with orthodontists in Australia (Meade and Weir 2022). Furthermore, issues regarding IPR timing and amount were reported by respondents as areas that always or mostly required changing from the initial DTP by 48%–56%, underlining the uncertainty of the role the procedure plays in CAT. Further research is required to determine whether the range of tools reportedly used by the respondents to carry out IPR is associated with the variety of responses related to the required frequency of IPR in the additional aligner/refinement plans.

The respondents reported that they prescribed a thermoplastic retainer in the maxilla and a combined bonded retainer and thermoplastic retainer in the mandible most frequently. This corresponded to the responses prescribed retainers following comprehensive fixed appliance treatment and CAT observed among orthodontists in Australia in recent surveys (Meade and Dreyer 2019; Meade and Weir 2022).

Over half of the respondents indicated that patients always, mostly, or sometimes reported difficulty in being compliant with CAT wear protocols. This corresponded with almost half of the free text comments provided by the respondents, where concerns regarding compliance with aligner wear protocols were expressed. Poor compliance is likely to result in suboptimal treatment outcomes. A recent study, however, has indicated that the use of e‐reminders and digital monitoring may improve compliance rates among those undergoing CAT (Timm et al. 2022).

Over 80% of respondents who did not provide CAT as an option considered that they preferred to refer to the orthodontist for patient management was a major or moderate factor in their decision not to provide the option. This may be linked to the almost 60% of respondents in the present survey who reported that insufficient postgraduate education was a moderate or major factor in their decision not to provide CAT. It also reflected the findings in the limited available relevant literature expressed by general dentists about undertaking further CAT‐related education (Aacaashnathan et al. 2023; d'Apuzzo et al. 2019; Gao et al. 2024).

An additional limitation to the low response rate was the risk of recall bias, whereby respondents may have provided replies that did not align exactly with their CAT practices.

However, the present survey is among the first of its kind. It provides baseline data regarding a wide range of factors related to CAT practices among general dentists. In the absence of robust evidence regarding CAT, it can provide information for clinicians, patients, aligner providers, and researchers.

## 5 Conclusions

The findings of the present survey indicated that almost two in three of the respondents provided CAT and that Invisalign was the most used system. The majority used CAT combined with nonextraction treatment. A wide range of CAT practices were reported by respondents who provided CAT. Over eight out of the 10 respondents who did not use CAT reported that the factor that mostly or moderately influenced their decision in this regard was a preference to refer to an orthodontist for management.

## Author Contributions

M.J.M. and T.W. conceptualized and designed the study. M.J.M. supervised the conduct of the study, data collection, managed the data, and undertook statistical analysis. M.J.M. and T.W. accounted for the quality control and provided advice on data analysis. M.J.M. and T.W. drafted the manuscript as submitted and agreed to be accountable for all aspects of the work.

## Conflicts of Interest

The authors declare no conflicts of interest.

## Supporting information

Supporting information.

## Data Availability

The data that support the findings of this study are available from the corresponding author upon reasonable request.
